# Insomnia and hallucinations in the general population: Findings from the 2000 and 2007 British Psychiatric Morbidity Surveys^☆^

**DOI:** 10.1016/j.psychres.2016.03.055

**Published:** 2016-07-30

**Authors:** Bryony Sheaves, Paul E. Bebbington, Guy M. Goodwin, Paul J. Harrison, Colin A. Espie, Russell G. Foster, Daniel Freeman

**Affiliations:** a Department of Psychiatry, University of Oxford, Warneford Hospital, Oxford OX3 7JX, UK; b Division of Psychiatry, University College London, 67-73 Riding House Street, London W1W 7EJ, UK; c Sleep and Circadian Neuroscience Institute (SCNi), Oxford Molecular Pathology Institute, Sir William Dunn School of Pathology, South Parks Road, Oxford OX1 3RE, UK

**Keywords:** Insomnia, Hallucination, Psychosis, Schizophrenia, Epidemiology

## Abstract

Insomnia is common in people experiencing psychosis. It has been identified as a contributory cause of paranoia, but any causal relationship with hallucinations has yet to be established. We tested the hypotheses that insomnia i) has a cross-sectional association with hallucinations ii) predicts new inceptions of hallucinations and iii) that these associations remain after controlling for depression, anxiety, and paranoia. Data from the second (2000, N=8580) and third (2007, N=7403) British Psychiatric Morbidity Surveys were used to assess cross-sectional associations between insomnia and hallucinations. The 2000 dataset included an 18 month follow up of a subsample (N=2406) used to test whether insomnia predicted new inceptions of hallucinations. Insomnia was associated with hallucinations in both cross-sectional datasets. Mild sleep problems were associated with 2–3 times greater odds of reporting hallucinations, whilst chronic insomnia was associated with four times greater odds. Insomnia was also associated with increased odds of hallucinations occurring *de novo* over the next 18 months. These associations remained significant, although with smaller odds ratios, after controlling for depression, anxiety and paranoia. This is the first longitudinal evidence that insomnia is associated with the development of hallucinatory experiences. Effective treatment of insomnia may lessen the occurrence of hallucinations.

## Highlights

• Insomnia was associated with hallucinatory experiences in both the 2000 and 2007 datasets.
• Initial insomnia was associated with new hallucinatory experiences 18 months later.
• The above associations remained significant after controlling for negative affect and paranoia.

## 1 Introduction

Insomnia is a common psychological disorder: approximately one third of the adult general population report disrupted sleep (Ohayon, 2002, Morphy et al., 2007), and around 6–10% will meet diagnostic criteria for insomnia disorder (Ohayon, 2002, Walsh, 2004). The importance of sleep in psychotic illness is beginning to be recognised (for a review of the area see: Reeve et al., 2015). For example, sleep disturbance is a risk factor for first episode psychosis (Ruhrmann et al., 2010) and subsequent relapse (Birchwood et al., 1989). There is also evidence of sleep and circadian rhythm disruption in patients with schizophrenia (Wulff et al., 2012, Afonso et al., 2014). Of individual psychotic experiences, only the relationship between paranoia and insomnia has received sustained attention (Reeve et al., 2015). Insomnia is very common in patients with persecutory delusions (Freeman et al., 2009), and predicts new inceptions and the persistence of persecutory ideation (Freeman et al., 2012). The association between insomnia and paranoia is partially mediated by affective symptoms (Freeman et al., 2010). This has led to sleep disruption being targeted in treatment (Freeman et al., 2015b, Freeman et al., 2015a, Myers et al., 2011, Waite et al., 2016). Indeed sleep disruption (and the impact on daytime psychotic symptoms) is highlighted as a problem that patients themselves would value help with, and treating sleep disruption in this group has proved a popular approach (Waite et al., 2015a). Recent studies provide the first evidence that sleep disruption and hallucinations are cross-sectionally associated (Koyanagi and Stickley, 2015, Taylor et al., 2015, Sheaves et al., 2016) This study will add to this emerging literature by assessing the cross sectional and longitudinal association between insomnia and hallucinations.

There are plausible mechanisms that may link the occurrence of insomnia with hallucinations. A recent twin study of 4800 adolescents demonstrated that 60–71% of the covariance between insomnia and psychotic experiences was attributable to additive genetic influences (Taylor et al., 2015). Non-shared environment accounted for the remaining 29–40% of covariance (Taylor et al., 2015). These links decreased, though remained significant after controlling for negative affect (Taylor et al., 2015). A specific association between insomnia and hallucinations was also investigated. A phenotypic correlation of .37 was reported between insomnia and hallucinations. The bivariate heritability (proportion of the association between these two phenotypes attributable to additive genetic effects) was .63, whilst the bivariate non-shared environment was .37 (Taylor et al., 2015).

Enhanced dopaminergic activity mediated via dopamine D2 receptors may underlie both positive symptoms of psychosis and increased wakefulness (Monti and Monti, 2005). Moreover, reducing sleep duration (i.e. sleep deprivation) can lead to anomalies of experience with close similarities to auditory hallucinations (Luby et al., 1960, West et al., 1962). It is highly likely that affective symptoms will mediate the association between insomnia and hallucinations, as shown for insomnia and paranoia (Freeman et al., 2010). Insomnia substantially increases the risk of affective disturbance (Baglioni et al., 2011) and insomnia treatment trials provide preliminary evidence for a causal relationship with affective symptoms (Manber et al., 2008, Espie et al., 2012). In turn, affective disturbance is extremely common in people experiencing clinical hallucinations and has long been recognised in cognitive models of psychosis (Morrison, 1998, Garety et al., 2001, Beck and Rector, 2003). In Beck and Rector's (2003) cognitive model, hallucinations manifest in part due to affective disturbance decreasing the threshold for hypervalent (or ‘hot’) cognitions to be experienced as identical to externally generated sounds (auditory hallucinations). In support of their association, there is evidence that affective symptoms trigger the onset (Slade, 1973) severity (Norman and Malla, 1991; Smith et al., 2006) and persistence of hallucinations (Escher et al., 2002, De Loore et al., 2011).

Psychotic experiences are not confined to patient groups, but also commonly occur in the general population (Johns et al., 2004, van Os et al., 2009). For example, 28.4% of a US community sample endorsed one or more Psychosis Screening Questionnaire (PSQ) items (Bebbington and Nayani, 1995), currently, whilst only 0.2–0.7% of the sample met diagnostic criteria when assessed by a clinician (Kendler et al., 1996). The persistence of these psychotic experiences over time increases the risk of transition to clinical psychosis (Dominguez et al., 2009). Nationally representative surveys can provide large-scale epidemiological tests of the associations of psychotic experiences with potential causal factors (Freeman et al., 2012, Marwaha et al., 2014). The current study seeks to establish from nationally representative UK survey data whether insomnia and hallucinatory experiences are associated. The following hypotheses were tested. i) Insomnia will be associated with the occurrence of hallucinatory experiences ii) sleep difficulties will predict the emergence of new hallucinatory experiences and iii) that these associations remain significant (albeit smaller) after controlling for affective symptoms (depression and anxiety) and paranoia.

## 2 Method

### 2.1 Setting and design

The second and third British adult psychiatric morbidity surveys aimed to assess the prevalence of mental health problems in adults living in private households. People aged 16–74 living in England, Wales and Scotland were included in the 2000 survey (*N*=8580, response rate=70%) and adults aged 16+ living in England were included in the 2007 survey (*N*=7403, response rate=57%). In both surveys the households were drawn from the small user Postal Address File. This includes all delivery points receiving less than 50 pieces of mail per day. Postal sectors were selected with a probability proportional to size. One person was selected per household. In 2000 the Kish grid method systematically selected one individual and in 2007, the individual was selected randomly. Two phases of interviews were completed for each single time point; phase one included structured assessments and screening measures, phase two was completed by clinically trained interviewers. Cases of psychotic disorder were identified in both surveys. Participants were screened for possible psychosis during phase 1, a process that included the PSQ (Bebbington and Nayani, 1995). They were invited for a phase 2 assessment of psychosis if they met one or more of the following criteria:

• Currently on antipsychotic medication.
• Hospital admission for mental health problems in the past 3 months.
• Positive response to question 5a on the PSQ (relating to auditory hallucinations).
• A self-reported diagnosis of psychotic disorder or of symptoms suggestive of it.

Participants not meeting any of these criteria were assumed not to have psychosis. For people interviewed in phase 2, the diagnosis of psychosis was based on the Schedule for Clinical Assessment in Neuropsychiatry (SCAN) (WHO, 1999). The category of “probable psychosis” included those diagnosed at SCAN interview, together with people who were not interviewed with SCAN, but met two of the above screening criteria.

In the 2000 survey, a sub-sample (*N*=2406) was interviewed 18 months following their initial interview to provide longitudinal data. Respondents to the 2000 survey were split into three groups: those with an identified mental disorder, those without a diagnosis but endorsing some symptoms of common mental disorders, and those reporting no symptoms. All those in sample groups one and two were included in the follow up, and 1 in 5 of the third group were interviewed, in order to avoid over-representation of this latter group. Further details regarding survey design can be found in previous methodological reports (Singleton et al., 2000; McManus et al., 2009).

The presence of hallucinations over the past 12 months was assessed using two items from the PSQ (Bebbington and Nayani, 1995). H1: Have there been times when you heard/saw things that other people couldn't? (PSQ5, yes/no) and H2: Did you at any time hear voices saying quite a few words or sentences when there was no one around that might account for it? (PSQ5a, yes/no). The full PSQ had a sensitivity of 96.9% and a specificity of 95.3% when compared with a clinical interview (Schedules for Clinical Assessment in Neuropsychiatry; Wing et al., 1990),.

Three levels of insomnia were calculated from items from the Clinical Interview Schedule Revised (CIS-R; Lewis et al., 1992), as in previous studies (Freeman et al., 2010):

I1: Endorsement of sleep difficulties over the past month (D1).

I2: Insomnia of moderate severity: Sleep difficulties (I1) for at least three nights per week (D3) and taking at least one hour to attain sleep (either initially attaining sleep, or returning to sleep; D5).

I3: Chronic insomnia: Insomnia of moderate severity (I2), which has lasted at least six months (D10) and reports of tiredness (B1).

Chronic insomnia is broadly representative of diagnostic criteria for Insomnia Disorder: a difficulty initiating or returning to sleep, which occurs at least three nights per week, for at least three months, with reports of daytime impairment (American Psychiatric Association, 2013).

A dimensional insomnia variable was calculated for longitudinal analysis in order to increase power for analysing this smaller sample. This summed CIS-R items assessing difficulties getting to or staying asleep (D1), number of nights with a sleep problem (D3), how much time spent getting to sleep (D5), number of nights spent longer than three hours trying to sleep (D6), early morning waking (D7), length of time experiencing sleep problems (D10) and reports of tiredness (B1). The score could range from 0 to 14.

Adjustments were made for current depression and anxiety using CIS-R symptom count scores (DVG11 and DVJ12). These measured frequency, duration, severity and time since onset of both anxiety and depression in the week prior to interview. Possible scores lay between zero and four. Adjustments for paranoia used an item from the PSQ: Have there been times when you felt that people were deliberately acting to harm you or your interests? (PSQ3a).

### 2.2 Analysis

The data were analysed using the Statistical Package for the Social Sciences (SPSS, version 19). All datasets were weighted to ensure they were representative of the national population by accounting for sampling rates, probability of selecting respondents, age, sex and region. Further weights were applied to the longitudinal dataset to account for the differential selection for follow-up interview. Binary logistic regression was used to assess the association between insomnia and hallucinations using three stages of analysis. Stage one reports unadjusted odds ratios, stage two adjusted for depression and anxiety, and stage three adjusted for depression, anxiety and paranoia. These adjustments were made in the light of already established associations linking insomnia with affective symptoms (Johnson et al., 2006) and paranoia (Freeman et al., 2010), both of which commonly co-occur with hallucinations. 95% confidence intervals (C.I.) are reported for the odds ratios.

## 3 Results

Within the 2000 cross-sectional dataset 3852 were male (weighted=46.8%) 4728 were females (weighted=53.2%). The age ranged from 16 to 74, the mean weighted age was 43.87 (standard error=0.23). In the 2007 cross-sectional dataset 3197 were male (weighted 48.6%) and 4206 were female (weighted 51.4%). The age ranged from 16 to 95, mean weighted age was 46.35 (standard error=.03). In the longitudinal dataset 1020 survey respondents were male (weighted 49.3%) and 1386 were female (weighted 50.7%). The age ranged from 16 to 74, mean weighted age was 43.44 (standard error=.49).

The prevalence of insomnia and hallucinatory experiences (H1 and H2) across both the 2000 and 2007 datasets is reported in Table 1. Probable psychosis was present in 60 cases in the 2000 survey (weighted 0.5%) and 40 cases (weighted 0.4%). Table 2 reports the endorsement of sleep disruption and hallucination variables in those with probable psychosis.

### 3.1 The cross-sectional association between insomnia and hallucinatory experiences

Table 3 presents the associations between insomnia and hallucinatory experiences, from both the 2000 and 2007 datasets. Experiencing sleep difficulties over the past month (I1) increased the weighted prevalence of hallucinatory experiences (H1) from 2.9–6.6% (2000 dataset) to 3.1–6.3% (2007 dataset). The experience of chronic insomnia (I3) increased the prevalence of hallucinatory experiences (H1) from 3.8% to 11.5% (2000) and from 3.8% to 10.8% (2007). Similarly, sleep difficulties increased the weighted prevalence of hearing voices saying several words or sentences (H2) from 0.5% to 1.5% (2000) and from 0.3% to 1.8% (2007). Chronic insomnia increased the weighted prevalence of endorsing H2 from 0.7% to 3.1% (2000) and from 0.7% to 3.3% (2007).

Associations between each of the three levels of insomnia and each of the two levels of hallucinatory experience were significant prior to adjustments. In general, the odds ratios (ORs) increased pari passu as severity of both insomnia and hallucinatory experiences increased. For example, sleep difficulties in the month prior to assessment were associated with a significant increase of odds for seeing or hearing things that others couldn't (H1; 2000 OR=2.36, 2007 OR=2.12). Chronic insomnia was associated with a significant increase of odds for hearing voices saying quite a few words or sentences (H2; 2000 OR=4.41, 2007 OR=4.83). As expected, adjusting for depression, anxiety and paranoia decreased the size of all ORs. Nevertheless, all but one association remained significant at alpha level .05, following these adjustments. The exception was chronic insomnia (I3) and hearing voices saying quite a few words or sentences (H2), which became non-significant (*p*=.25).

### 3.2 The longitudinal association between insomnia and the onset of new hallucinatory experiences

This analysis excluded those who endorsed hallucinatory experiences at time one, in order to assess insomnia as a risk factor for new hallucinatory experiences. The ORs for the dimensional measure of insomnia and its association with the two levels of hallucinatory experiences (H1 and H2) are presented in Table 4. Insomnia at time one is significantly associated with new hallucinatory experiences eighteen months later. Results remained significant after adjustment for baseline levels of anxiety, depression, and paranoia. The odds ratios in Table 4 reflect a one-point increase on the insomnia dimensional scale. A ten-point increase on the insomnia scale is thus associated with an odds ratio of 17.3 for hearing voices saying several words (1.33 raised to the power of 10).

## 4 Discussion

This study used two large nationally representative datasets to study, for the first time, the cross-sectional and longitudinal associations between insomnia and hallucinatory experiences. In line with our hypotheses, insomnia was associated with hallucinatory experiences in both the 2000 and 2007 cross-sectional datasets. Importantly, this association was not wholly attributable either to negative affect (depression and anxiety) or to co-morbid paranoia. As would be expected, controlling for both negative affect and paranoia reduced the size of the associations, but the direct relationship remained significant. The advantage of studying two datasets is that we have built-in replication. The second key finding was that initial insomnia was associated with increased inceptions of hallucinatory experiences eighteen months later. It was only possible to study this effect in one of the surveys. However the importance of this result lies in its indication that insomnia is not simply a consequence of hallucinations. Whilst it is of course possible that insomnia results from hallucinations, the current results reveals that insomnia may be a risk factor for the occurrence of hallucinations.

These results add weight to the view that insomnia might have a contributory causal role in the onset of psychotic experiences (Freeman et al., 2012). A next step in testing this hypothesis is to treat insomnia and assess the impact on psychotic experiences (an approach termed an interventionist model of causality (Kendler and Campbell, 2009)). A very large (over 2000 participants) randomised controlled trial (RCT) of CBT for insomnia (i.e. the recommended treatment for persistent insomnia; Morin and Benca, 2012, National Institute for Health and Care Excellence 2015. Managing long term insomnia (> 4 weeks) [WWW Document]. NICE Clinical Knowledge Summary. URL http://cks.nice.org.uk/insomnia#!scenario:1 (accessed 5.11.15)..) is currently underway (Freeman et al., 2015b). This study will examine the impact of improved sleep on psychotic like experiences (and other mental health difficulties) at an age when they typically emerge. The intervention will be offered digitally (via computer and smartphone) which is a cost-effective approach. Given that the persistence of psychotic experiences (Dominguez et al., 2009), and the presence of sleep disturbance (Ruhrmann et al., 2010) constitute risk factors for clinical psychosis, the impact of treating sleep on these sub-clinical phenomena is of both clinical and theoretical interest.

There are clear limitations to the current study. While epidemiological studies enable the testing of large numbers of people, they limit the depth of the assessments. The number of people endorsing hallucinations was small in each dataset. Thus, the power to study new inceptions of hallucinations was particularly limited and we could not test whether the few people who had hallucinations at baseline went on to develop insomnia (i.e. to test the reverse hypothesis). Our view is that there is likely to be a reciprocal relationship between insomnia and psychotic experiences such as hallucinations. This bidirectional causal relationship requires testing in adequately powered studies. Moreover, epidemiological studies leave open the possibility that confounding and other related but distinct variables (e.g. past or family history of sleep and psychiatric disorders) may provide better explanations for the reported associations between insomnia and hallucinations. While we controlled for two variables where there were *a priori* reasons for doing so (negative affect and paranoia), experimental manipulations are needed to rule out whether other factors, (e.g. substance use) influence the relationship. Finally, while it is tempting to assume that inferences from the general population apply to clinically relevant first episodes of psychosis, it will be important to confirm this in appropriately designed studies of high risk individuals.

Notwithstanding these caveats, the current study establishes a consistent cross sectional association between insomnia and hallucinations, and indicates that insomnia might be one risk factor for inceptions of hallucinations. These associations mirror those found between insomnia and paranoia. The clinical implication is that treating sleep disturbance might alleviate a risk factor common to both hallucinations and persecutory delusions.

## Conflict of interest

**BCS** provides clinical support to Sleepio Ltd.

**GMG** holds shares in P1vital and has served in the last 2 years as consultant, advisor or CME speaker for AstraZeneca, Abbvie, Cephalon/Teva, Convergence, Eli Lilly, GSK, Lundbeck, Medscape, Merck, Otsuka, P1Vital, Servier, Sunovion, Takeda.

**CAE** holds shares in Sleepio/ Big Health Ltd and has served in the last 2 years as consultant, advisor or CME speaker for Boots Pharmaceuticals, UCB, and Novartis.

All other authors have no conflict of interest.

## Figures and Tables

**Table 1:** The frequency and weighted prevalence of sleep disruption and hallucinations in the total 2000 and 2007 datasets.

	**2000**		**2007**	
	**Frequency**	**Weighted prevalence (%)**	**Frequency**	**Weighted prevalence (%)**
**Sleep difficulties**	3380	38.0	3096	39.4
**Insomnia**	1120	11.9	1123	13.7
**Chronic insomnia**	623	6.6	638	7.6
**H1**	371	4.3	323	4.3
**H2**	82	0.9	68	0.9

H1=‘In the past year, have there been times when you heard or saw things that other people couldn't’. H2=‘Did you at any time hear voices saying quite a few words or sentences when there was no one around that might account for it?’

**Table 2:** The frequency and weighted prevalence of sleep disruption and hallucinations in those with probable psychosis.

	**2000**		**2007**	
	**Frequency**	**Weighted prevalence (%)**	**Frequency**	**Weighted prevalence (%)**
Sleep difficulties	40	66.5	25	67.5
Insomnia	22	36.3	13	33.8
Chronic insomnia	21	34.0	11	29.6
H1	27	49.1	19	47.4
H2	25	45.6	15	37.1

H1=‘In the past year, have there been times when you heard or saw things that other people couldn't’. H2=‘Did you at any time hear voices saying quite a few words or sentences when there was no one around that might account for it?’

**Table 3:** The cross-sectional relationship between insomnia and hallucinatory experiences in the 2000 and 2007 datasets.

	Unadjusted			Adjusting for depression and anxiety				Adjusting for depression, anxiety and paranoia		
	Odds ratio	*P-*value	95% C. I.	Odds Ratio	*P-*value	95% C. I.		Odds ratio	*P-*value	95% C. I.
**2000 dataset**										

H1: ‘In the past year, have there been times when you heard or saw things that other people couldn't’										
I1: Sleep difficulties	2.36	<0.001	1.84–3.03	1.76	<0.001	1.35–2.29		1.65	<0.001	1.27–2.16
I2: Insomnia	2.69	<0.001	2.04–3.55	1.71	0.001	1.25–2.34		1.61	0.003	1.17–2.20
I3: Chronic insomnia	3.28	<0.001	2.40–4.50	1.93	<0.001	1.35–2.78		1.86	0.001	1.30–2.66

H2: ‘Did you at any time hear voices saying quite a few words or sentences when there was no one around that might account for it?’										
I1: Sleep difficulties	3.32	<0.001	2.00–5.52	1.97	0.016	1.13–3.42		1.78	0.044	1.02–3.11
I2: Insomnia	4.14	<0.001	2.56–6.68	1.98	0.017	1.13–3.47		1.82	0.040	1.03–3.21
I3: Chronic insomnia	4.41	<0.001	2.67–7.29	1.83	0.033	1.05–3.18		1.74	0.047	1.01–3.00
									
**2007 dataset**										

H1: ‘In the past year, have there been times when you heard or saw things that other people couldn't’										
I1: Sleep difficulties	2.12	<0.001	1.65–2.72	1.66	<0.001	1.27–2.18		1.49	0.005	1.13–1.96
I2: Insomnia	2.56	<0.001	1.92–3.42	1.78	0.001	1.28–2.48		1.61	0.006	1.15–2.25
I3: Chronic insomnia	3.07	<0.001	2.18–4.34	2.03	0.001	1.36–3.01		1.72	0.011	1.13–2.61

H2: ‘Did you at any time hear voices saying quite a few words or sentences when there was no one around that might account for it?’										
I1: Sleep difficulties	5.22	<0.001	2.74–9.96	3.05	0.003	1.47–6.32		2.69	0.010	1.27–5.66
I2: Insomnia	7.08	<0.001	4.12–12.16	3.56	<0.001	1.76–7.17		3.13	0.002	1.54–6.36
I3: Chronic insomnia	4.83	<0.001	2.65–8.81	1.94	0.071	0.95–3.96		1.55	0.245	0.74–3.27

**Table 4:** The longitudinal associations between insomnia at time 1 and experience of hallucinatory experiences 18 months later.

Predictor variable	*n*	OR	*P-*value	95% CI
H1: ‘In the past year, have there been times when you heard or saw things that other people couldn't’				
Insomnia T1 (dimensional score)	2251	1.17	0.004	1.05–1.30
Insomnia controlling for T1 depression and anxiety	2251	1.14	0.010	1.03–1.26
Insomnia controlling for T1 depression, anxiety and paranoia	2251	1.13	0.013	1.03–1.25

H2: ‘Did you at any time hear voices saying quite a few words or sentences when there was no one around that might account for it?’				
Insomnia T1 (dimensional score)	2375	1.33	0.003	1.10–1.60
Insomnia controlling for T1 depression and anxiety	2375	1.28	0.014	1.05–1.57
Insomnia controlling for T1 depression, anxiety and paranoia	2375	1.28	0.027	1.03–1.58

NB: The odds ratios in this table reflect a one-point increase on the insomnia dimensional scale. A ten-point increase on the insomnia scale is thus associated with an odds ratio of 17.3 for hearing voices saying several words (1.33 raised to the power of 10).
